# Speech understanding in noise with the Roger Pen, Naida CI Q70 processor, and integrated Roger 17 receiver in a multi-talker network

**DOI:** 10.1007/s00405-015-3643-4

**Published:** 2015-05-16

**Authors:** Geert De Ceulaer, Julie Bestel, Hans E. Mülder, Felix Goldbeck, Sebastien Pierre Janssens de Varebeke, Paul J. Govaerts

**Affiliations:** The Eargroup, Antwerp-Deurne, Belgium; Advanced Bionics AG, Clinical Research International, Staffa, Switzerland; Phonak Communications AG, Murten, Switzerland; ENT Department, Jessa Hospital, Hasselt, Belgium; University of Antwerp, Antwerp, Belgium; University of Ghent, Ghent, Belgium

**Keywords:** Cochlear implant, FM, Speech perception, Noise, Frequency modulation, Assistive listening

## Abstract

Roger is a digital adaptive multi-channel remote microphone technology that wirelessly transmits a speaker’s voice directly to a hearing instrument or cochlear implant sound processor. Frequency hopping between channels, in combination with repeated broadcast, avoids interference issues that have limited earlier generation FM systems. This study evaluated the benefit of the Roger Pen transmitter microphone in a multiple talker network (MTN) for cochlear implant users in a simulated noisy conversation setting. Twelve post-lingually deafened adult Advanced Bionics CII/HiRes 90K recipients were recruited. Subjects used a Naida CI Q70 processor with integrated Roger 17 receiver. The test environment simulated four people having a meal in a noisy restaurant, one the CI user (listener), and three companions (talkers) talking non-simultaneously in a diffuse field of multi-talker babble. Speech reception thresholds (SRTs) were determined without the Roger Pen, with one Roger Pen, and with three Roger Pens in an MTN. Using three Roger Pens in an MTN improved the SRT by 14.8 dB over using no Roger Pen, and by 13.1 dB over using a single Roger Pen (*p* < 0.0001). The Roger Pen in an MTN provided statistically and clinically significant improvement in speech perception in noise for Advanced Bionics cochlear implant recipients. The integrated Roger 17 receiver made it easy for users of the Naida CI Q70 processor to take advantage of the Roger system. The listening advantage and ease of use should encourage more clinicians to recommend and fit Roger in adult cochlear implant patients.

## Introduction

In everyday listening situations, the presence of reverberation and background noise can make it difficult to understand an individual speaker. Moreover, as sound travels away from the source it reduces in intensity, while the background noise remains relatively constant. Hence the signal-to-noise ratio (SNR) decreases [1]. The combination of reverberation, background noise, and distance from the speaker results in poor listening conditions, even for normally hearing individuals, but the impact is even greater on the hearing impaired [2].

Wireless microphones, also known as frequency modulated (FM) systems, are assistive listening devices designed to help hearing impaired individuals in these challenging listening conditions. They consist of a microphone placed near the speaker’s mouth, which picks up the sounds, converts them to an electrical waveform, and transmits the signal directly to a receiver worn by the listener. By acquiring the signal at or near the source, the negative effects of ambient noise, as well as those of distance are reduced and consequently the SNR at the listener’s ear is improved.

In adults with mild to severe hearing loss, strong evidence shows that the use of FM systems results in better speech understanding in noise than the use of hearing aids alone [3–5]. However, the adoption rate of FM systems by this population is low, possibly due to cost, esthetics, or lack of counseling [3, 5].

FM systems can also benefit adults using cochlear implants (CI) resulting in significant improvements in speech perception in noise and improvements in sound clarity, ease of listening, and sound quality [6, 7]. Fitzpatrick et al. [8] conducted a purely subjective evaluation, designed to document FM use in everyday situations in a diary, and showed that CI users do perceive that they benefit from the FM when at a distance from the speaker or in noise. Nonetheless, the physical size of the device and the complexity of connecting it to the CI did deter some subjects from using the FM, even when they recognized that some speech perception improvement could be gained.

Fixed gain FM receivers apply one gain value to all incoming signals, typically +10. Dynamic gain offers the ability for this parameter to be altered automatically, depending on the speech and noise content of the input signal. In the Phonak MLxi receiver (Phonak AG, Switzerland), a low gain of +10 is used when the ambient noise level is below 57 dB SPL increasing to +24 at a noise level of 75 dB SPL. In a study of two groups of CI users using the Phonak inspiro FM Transmitter and the MLxi (adaptive) or MLxS (fixed) FM receivers, Wolfe et al. [9] found that the Dynamic FM resulted in significantly better performance for sentences in noise compared to the traditional FM. Significant improvements in sentences in noise were also observed for Dynamic FM when used in combination with hearing aids at higher noise levels [10].

A further study by Wolfe et al. [11] looked at the latest technology in this area—digital transmission of the signal between the transmitter and receiver. This system no longer uses frequency modulation, and therefore technically speaking can no longer be referred to as an FM system. The Phonak Roger system (Mülder, Roger: The new wireless technology standard, Phonak Insight 2013) features an adaptive gain adjustment similar to the Phonak MLxi system, but using a digital signal transmission and digital signal processing to manipulate the gain increases. This system was compared to the MLxS fixed gain receiver as well as to the MLxi in a classroom-like setup with a single talker [11]. Subjects were16 CI recipients fitted with an Advanced Bionics Harmony speech processor and 21 recipients fitted with a Cochlear CP810 processor. The Phonak inspiro transmitter was used, and the FM receivers were coupled to the CI speech processors using the iConnect FM earhook and the Europlug adaptor, respectively. Speech perception results were significantly better in all the FM conditions compared to the no-FM conditions. At the highest noise levels, the Roger system provided significantly better speech perception compared to either the fixed gain MLxS or the adaptive gain MLxi receivers. This additional advantage over the analog adaptive gain system may be due to the wider bandwidth provided by the digital system or to the clearer signal transmission. The authors note that the results of the study can only be applied to the Harmony and CP810 processors, although a similar study also showed that the Roger system was superior to the MLxi and MLxS at the higher noise levels with a MED-EL Opus2 speech processor [12].

The Naída CI Q70 speech processor is the latest in speech processor technology offered by Advanced Bionics. Recent results reported by Wolfe [13] demonstrate that Roger provides significant benefit for understanding speech in high levels of noise when used with the integrated Roger 17 receiver on the Naida CI Q70 in a simulated classroom environment with a single talker.

## Objectives

The primary objective of this study was to determine the speech perception advantage gained in noise with the Roger Pen transmitter and the Naída CI Q70 speech processor with integrated Roger 17 receiver. While most previous research with FM systems has focused on the classroom situation with a single talker, the present study was setup to test the Roger system in a group conversation situation, in a simulated restaurant environment with multiple talkers. In this situation, multiple Roger Pens, one for each talker, can be used in a network configuration where more than one microphone are able to transmit to the receiver individually. To compare with a fair alternative, subjects were also evaluated in the same configuration with just one Roger Pen lying in the middle of the table. This single-transmitter situation would currently be the typical way a user would take advantage of the Roger Pen or any alternative wireless microphone system in a lively discussion with multiple speakers.

## Methods

### Study design

Twelve adults participated in this randomized, prospective, within-subjects repeated measures design study. The study was approved by the Ethical Board of Jessa Hospital (Hasselt, Belgium) on August 5th 2014, under the reference “14.53/ORL14.02.”

### Participants

Twelve post-lingually deafened adult subjects were randomly recruited from two sites, Eargroup (Antwerp) and Jessa Hospital (Hasselt), and were assessed at Eargroup during one acute session. All subjects were users of CII/HiRes 90K cochlear implant (unilaterally or bilaterally implanted). Demographics are listed in Table 1.

**Table 1:** Subject demographic data including the type of speech processor microphone used in the “no pen” condition and the clear voice gain settings

Subject ID	Age (years)	CI side	Duration of HL (years)	Duration of CI use (years)	Clear voice setting	Microphone
S1	29	Left	7	1	Medium	Tmic
S2	68	Left	0	1	Medium	Tmic
S3	35	Left	21	1	Medium	Tmic
S4	71	Left	14	0	Medium	Tmic
S5	61	Right	0	1	Medium	Tmic
S6	65	Left	0	1	Medium	Tmic
S7	36	Right	23	6	Medium	Processor mic
S8	63	Left	0	6	Off	Tmic
S9	56	Right	8	7	Medium	Processor mic
S10	71	Left	0	7	Off	Processor mic
S11	64	Left	1	1	Medium	Tmic
S12	26	Right	11	10	Medium	Tmic

If the subject was a new implant recipient, at least 3 months of Naída CI Q70 use was required at time of testing. Experienced implant users had used the Naída CI Q70 for at least 1 month. Concurrent participation in another study and difficulties additional to hearing impairment that would interfere with the study procedures were considered as exclusion criteria. Written informed consent was obtained from all subjects.

### Test setup

**Fig. 1:**
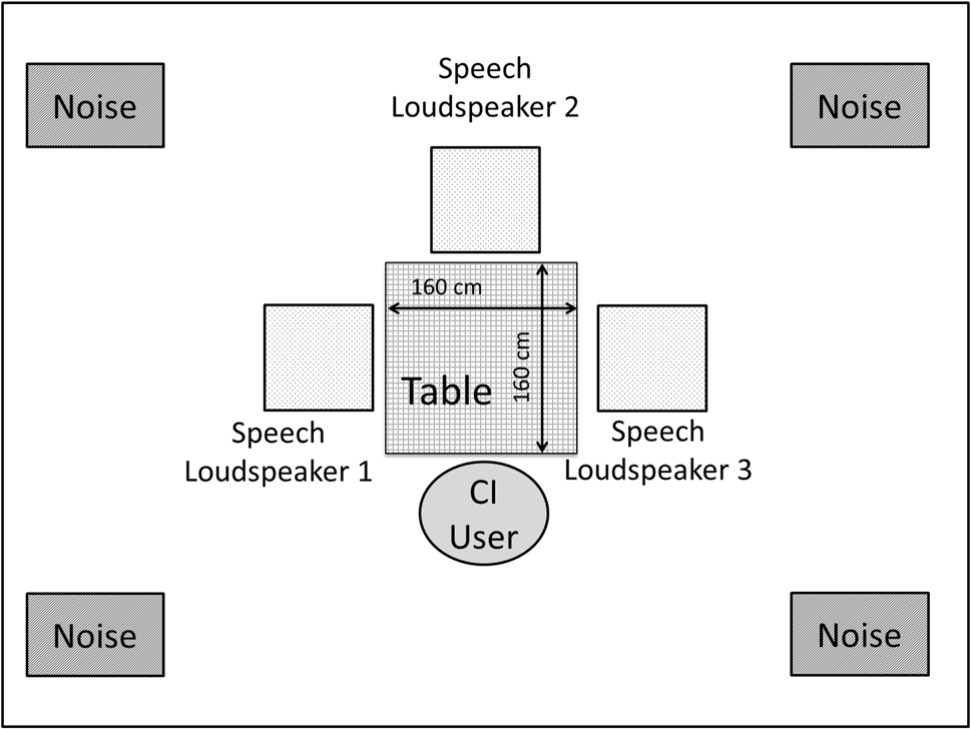
Test setup of the room showing speaker locations and distances. The CI user is seated at one side of a 160-cm square table with speech speakers positioned in the middle of the three other sides. Four noise speakers are positioned in the corners of the room

A test situation was created to simulate four persons having a meal in a noisy restaurant, one of them being the CI user (listener), while the three companions (talkers) were talking non-simultaneously. The three talkers were mimicked by three Fostex 6301B Personal loudspeakers placed around the listener as shown in Fig. 1. A diffuse field of multi-talker babble noise, acquired by recording samples from 100 people speaking in a canteen (http://spib.linse.ufsc.br/noise.html), was created by means of four Alesis Elevate noise generating speakers which were placed in the corners of the room.

All the speakers were connected to a PC via a Gigaport Soundcard. The A§E software platform (Otoconsult nv, Antwerp, Belgium), as detailed in Govaerts et al. [14], generated and controlled individual speech and noise presentation on all speakers and allowed random presentation of individual sentences on one of the three Speech speakers.

### Procedures and device fitting

Testing was done in three conditions: (1) without Roger Pen (condition “no pen”), (2) with one Roger Pen transmitter-microphone (condition “1 pen”), and (3) with three Roger Pens in a multi-talker network (MTN) (condition “3 pens”). In an MTN, each conversation partner wears his own wireless microphone. One ‘master’ microphone in the network controls which microphone is open at any one time, so only one microphone is active and all other microphones are muted. Voice activity steers the switching between microphones, which occurs automatically and quickly on a first come, first-served basis.

**Fig. 2:**
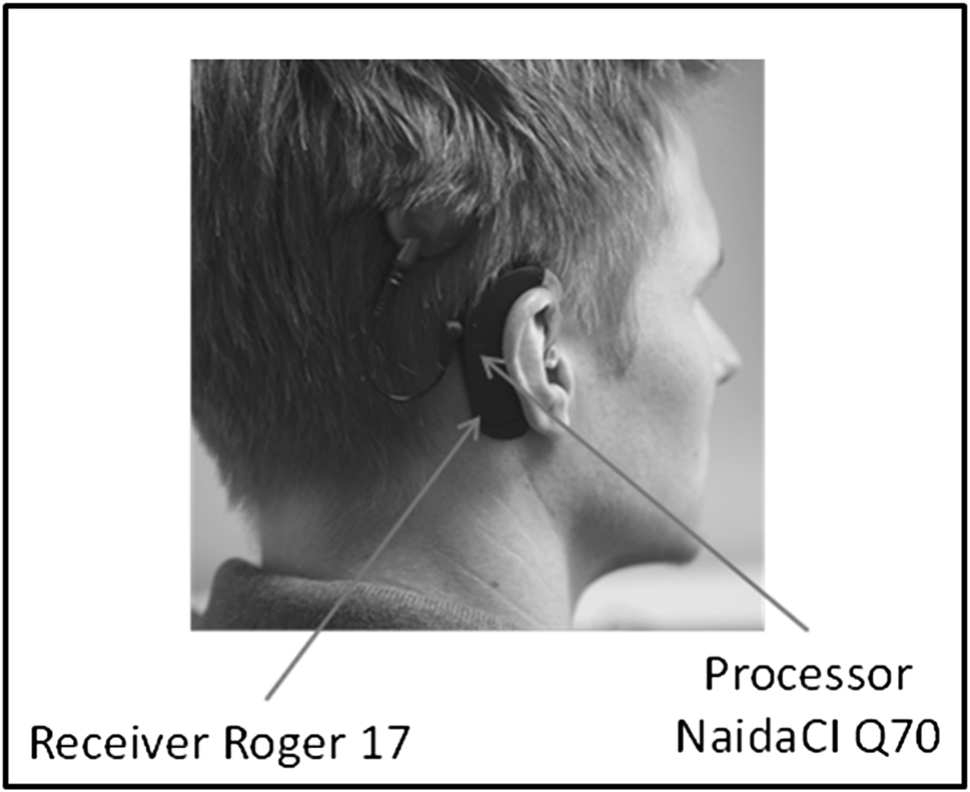
User wearing a Naida CI Q70 connected with the integrated Roger 17 receiver. The Roger 17 receiver is connected by sliding it over the bottom of the PowerCel 170 battery pack

For conditions two and three, the listener was wearing the Naída speech processor connected to a Roger 17 receiver (Fig. 2).

**Fig. 3:**
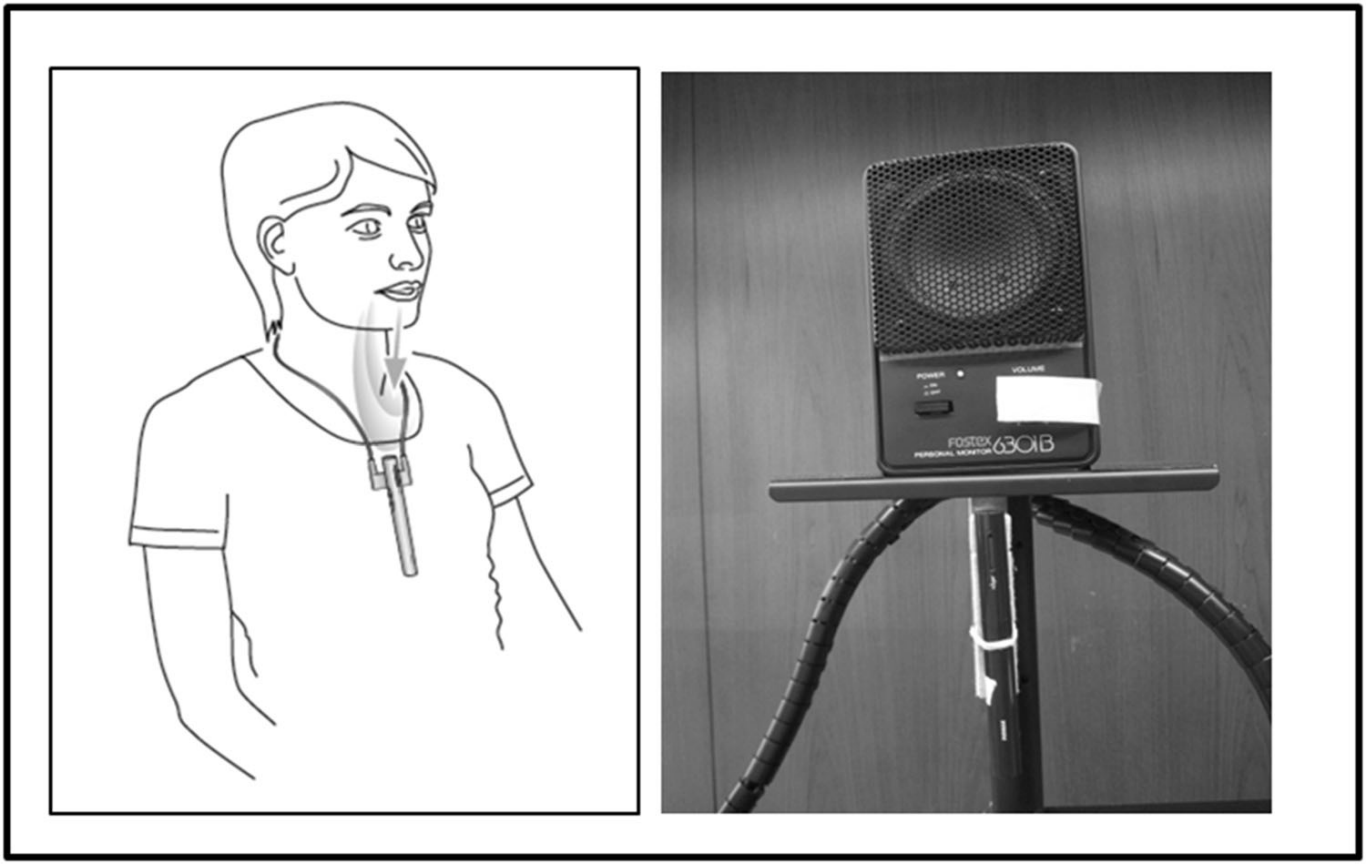
*On the left* a talker is wearing a Roger Pen around the neck with the Lanyard adjusted so that the pen is optimally placed (ideally 20 cm from the mouth). *On the right* side, this situation is transposed to the lab, where the talker is replaced by a loudspeaker, with the Roger Pen optimally placed at 20 cm to pick up the sound coming out of it

For the “1 pen” condition, a single Roger Pen transmitter was placed in the middle of the table. For the “3 pens” condition, each of the three talkers was equipped with a Roger Pen transmitter that was placed in front of each of the three loudspeakers at approximately 20 cm, at a necktie position relative to the speaker (Fig. 3).

All subjects had been fitted with the HiRes Optima (Sequential) sound processing strategy and an input dynamic range of 80 dB on their everyday processors. Program parameters were set according to the FOX target driven, computer-assisted approach described in Govaerts et al. [14, 15, 16].

For this study, each subject was provided with a new Naída CI Q70 CI processor for the purposes of testing only without any program changes. The Roger 17 receiver was connected to the processor through the Naída Powercel 170 auxiliary input. In the “no pen” condition, the CI recipient’s current “daily” program and microphone option were used. This microphone option could either be the T-mic or processor mic dependent on his/her preference [17]. For the “1 pen” and “3 pens” conditions, this “daily” program was modified by changing the signal input from 100 % microphone input to a 50:50 mix of the recipient’s current microphone option and Roger 17 on the aux input, as recommended by Wolfe and Schafer [7] and Advanced Bionics.

Roger automatically adjusted the gain depending on the noise level. In the “3 pens” condition, the Roger Pens all automatically worked in a beam-forming microphone mode. In the “1 pen” condition, with the Roger Pen lying horizontally on a table, the Roger Pen automatically selected an omnidirectional microphone mode for noise levels below 70 dB SPL. At higher ambient noise levels, the Roger Pen automatically switched to beam forming. Built-in hysteresis prevents frequent switching between omnidirectional and beam forming when the noise level hovers around 70 dB SPL.

Seven of the twelve subjects wore only one implant. The five bimodal users (CI + contralateral hearing aid) were instructed to take their hearing aid off during the testing. There was no bilateral Naída CI Q70 user included in the study.

### Outcome measures

Speech perception was tested using the Flemish sentences-in-noise test [18, 19]. This test consists of 36 lists of 10 sentences each, characterized by a varying number of target words. Scores were recorded as the percentage of the target words correctly repeated by the listener.

Individual sentences were presented randomly from one of the three Talker loudspeakers for each sentence in the list. The presentation level of the speech was fixed at 65 dB SPL measured at the listener’s head for each speaker individually. Multi-talker babble noise [20] was presented through the Noise speakers at fixed levels of 55, 60, 65, 70, 75, and 80 dB SPL. Hence, all listeners were tested in three conditions and at six different SNRs per condition. The order of conditions was randomized, and subjects were not aware of the testing condition (single-blinded trial).

The speech reception threshold (SRT) was calculated (by linear interpolation) as the SNR at which a 50 % score was obtained. The SRT constituted the primary study outcome, while the word score at each SNR was also computed and provided the secondary outcome measure.

### Statistics

Descriptive statistics were computed for SRT results and percent correct word scores. A normality test (Shapiro–Wilks) was run to decide if a parametric or non-parametric repeated ANOVA should be conducted to compare SRT in the three conditions. The secondary outcome measures were assessed by means of a two-way ANOVA with two repeated factors (SNR and condition). For each repeated ANOVA analysis, the sphericity assumption (equal sub-populations variances) was tested using Maulchy’s test, which led to run a correction for multivariate effects when necessary (Greenhouse-Geisser correction). Results were considered to be statistically significant for *p* values less than 0.05. All analyses were conducted with Statistica software (version 9.1, Statsoft Corporation).

## Results

Prior to taking the tests in noise, subjects were systematically assessed in quiet with a list of sentences delivered at 65 dB SPL. Scores ranged from 67.5 to 100 % with an average of 93.4 %.

Individual SRTs are listed in Table 2. Performance improved progressively for 10 out of 12 subjects as they progressed from the “no pen” to “3 pen” conditions. All subjects showed a negative SRT in the “3 pen” condition and a large improvement over the “no pen” condition.

**Table 2:** Individual SRTs for each subject, in dB, in each of the three test conditions

Subject ID	No pen	1 pen	3 pens
S1	5.9	1.7	−12.4
S2	7.1	4.7	−11.3
S3	4.8	6.7	−6.1
S4	6.2	5.6	−7.6
S5	3.2	−0.4	−10.5
S6	3.5	1.1	−9.4
S7	3.5	1.5	−12.5
S8	3.5	2.5	−11.1
S9	0.4	1.9	−9.5
S10	7.9	4.8	−5.9
S11	7.1	5.0	−12.3
S12	6.9	4.9	−8.7

A lower score indicates better performance

**Fig. 4:**
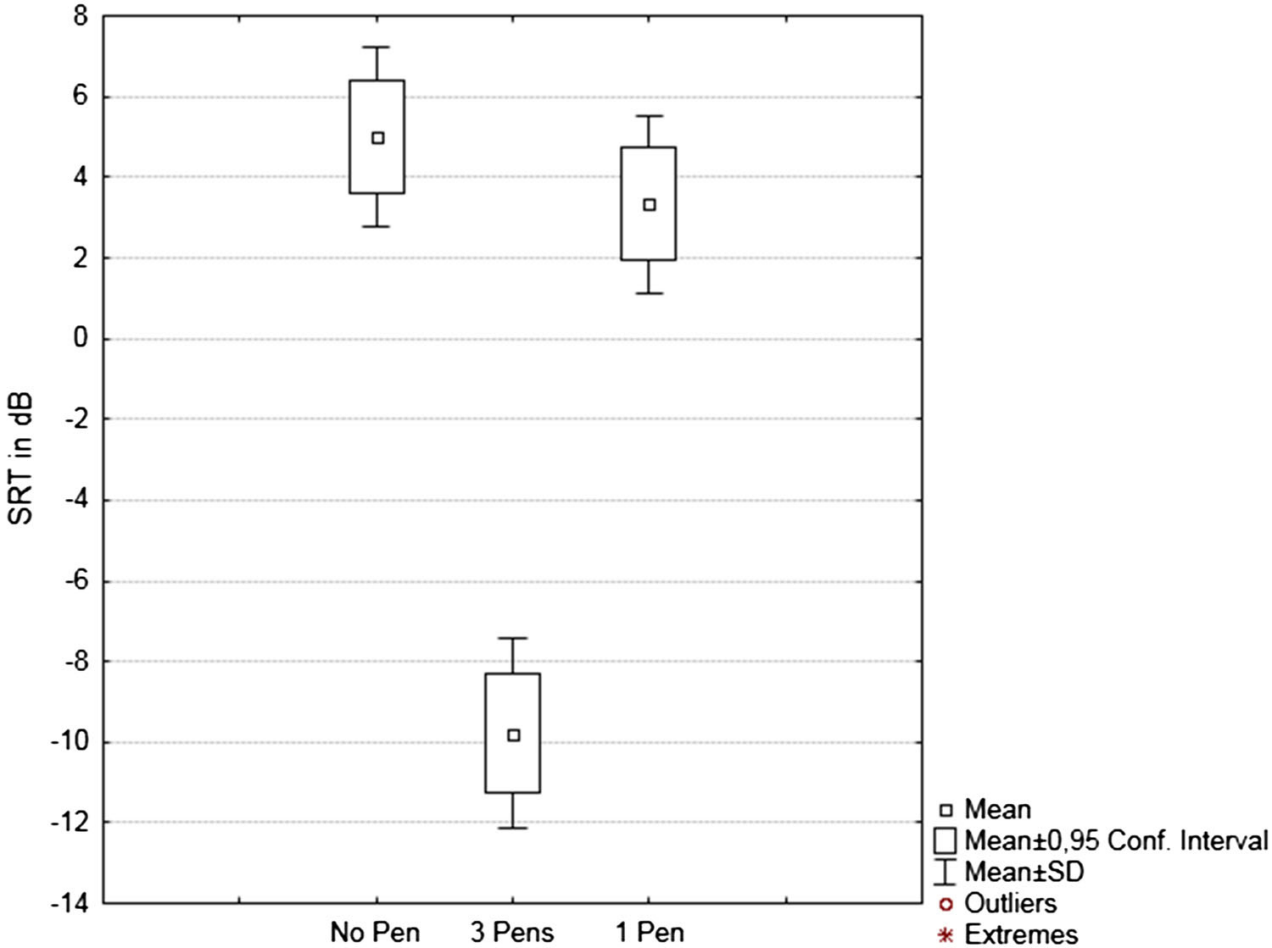
*Box plots* of SRT in the three conditions, with no pen, with one pen placed in the middle of the table, and with three pens placed at each speech loudspeaker. Mean values for SRT in dB are shown, with 95 % confidence intervals and SD

Shapiro-Wilks tests were non-statistically significant for the 3 distributions (*p* = 0.31, *p* = 0.25 and *p* = 0.35 for “no pen”, “3 pens” and “1 pen”, respectively), and neither was Mauchly’s test for sphericity (*p* = 0.168). Hence a parametric one-way repeated ANOVA was used to assess differences between the three conditions. A significant difference was found for the whole model (*F*(2,22) = 276.32, *p* < 0.0001). Post hoc pairwise comparisons (Bonferroni correction) showed that there was a highly statistically significant (*p* < 0.0001) improvement in mean SRT from +5 dB with “no pen” and +3.3 dB with “1 pen” to −9.8 dB in the “3 pens” configuration. The mean difference of 1.7 dB between the conditions “no pen” and “one pen” was not statistically significant (*p* = 0.07). Box plots showing means, 95 % confidence intervals and SD in the three conditions are depicted on Fig. 4.

The secondary outcome measure was assessed with a 2-way repeated ANOVA with the factors condition (3 levels) and SNR (6 levels). Both main effects of condition and SNR were significant (condition: *F*(2,22) = 478.34, *p* < 0.0001, SNR: *F*(5,55) = 293.97, *p* < 0.0001), as was the interaction between them (condition × SNR: *F*(10,110) = 32.73, *p* < 0.0001).

Post hoc analysis showed statistically significant differences in averaged scores between the “3 pens” and “no pen” conditions at every SNR except −15 dB: 16.2 % at +10 dB SNR (*p* = 0.02), 40 % at +5 dB SNR (*p* < 0.001), 77.3 % at 0 dB SNR (*p* < 0.001), 78.6 % at −5 dB SNR (*p* < 0.001), 48.8 % at −10 dB SNR (*p* < 0.001), and 48.8 % at −10 dB SNR (*p* < 0.001). The mean difference in scores at −15 dB was 14.5 % but not statistically significant.

Similar post hoc comparisons between the “3 pens” and “1 pen” conditions showed that the following differences in average scores were highly statistically significant (*p* < 0.001): 26.1 % at +5 dB SNR, 70.9 % at 0 dB SNR, 78 % at −5 dB SNR, and 48.8 % at −10 dB SNR. The average differences in scores were 9.9 % for +10 dB SNR and 14.5 % for −15 dB SNR. Those differences were not statistically significant.

Post hoc analysis of the differences between the “no pen” and “1 pen” conditions showed that none was statistically significant. Notably, the largest mean difference was 13.9 %, obtained at an SNR of +5 dB (*p* = 0.15).

**Fig. 5:**
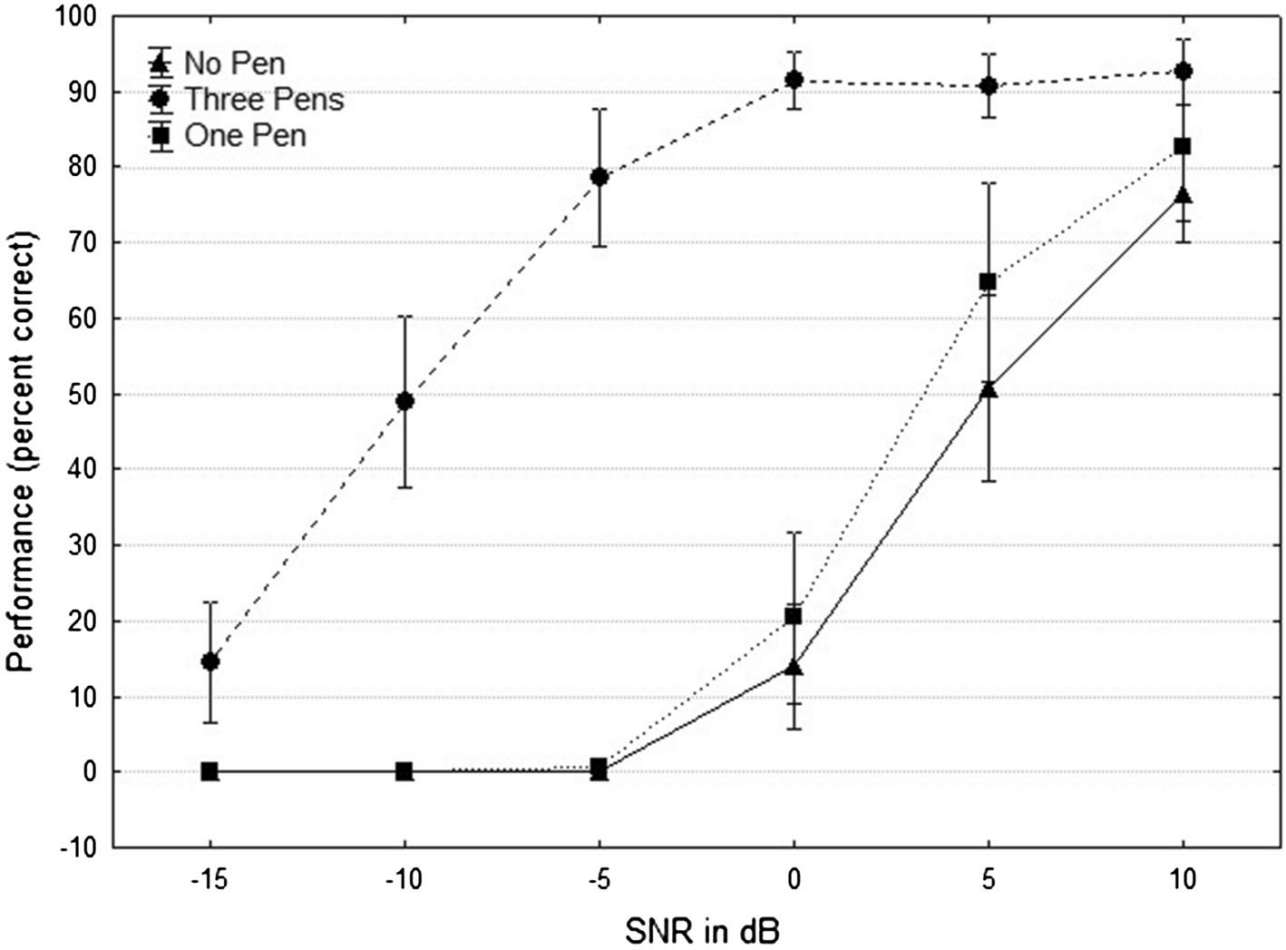
Graph showing average percent correct word scores in the three conditions (no pen, three pens, and one pen) at each tested SNR: from 10 to −15 dB by steps of 5 dB. *Error bars* show 95 % confidence intervals

Mean scores and 95 % confidence intervals in the three conditions are depicted in Fig. 5.

## Discussion

The study showed that, when using the Roger Pen in the MTN, there was a large statistically and clinically significant improvement in subject’s speech perception performance compared to either one pen or no pen. This is the first study to test a multiple talker network with adult CI users in a realistic everyday situation. Previous studies have focused on the use of single transmitters in a classroom situation with large distances between the talker and the listener (5–6 m), a scenario more representative of FM use in children at school [9, 11, 21]. The test setup created here was specifically designed to be relevant for adults and the situations in which that they would be more likely to use a Roger system, i.e., in small group conversations or discussions.

In contrast to the Wolfe et al. study with the Roger system and the Advanced Bionics Harmony processor [11], here the Roger receiver connected directly to the Naída CI Q70 speech processor in a new specially designed integrated unit. With the MTN, the average gain in SRT was 14.8 dB over the “no pen” condition and a 13.1 dB improvement over the “1 pen” condition, which represents a very large clinical advantage. To put this advantage into context, a typical restaurant has an ambient noise level of 70 dB SPL and conversational speech at around 65 dB SPL. At this SNR (−5 dB), subjects scored on average 80 % correct in the “3 pens” condition compared to 0 % in the “1 pen” or “no pen” conditions. The SRT scores were affected by ceiling effects in the “3 pens” condition and floor effects in the “1 pen” and “no pen” conditions, resulting in non-significant differences for the secondary outcome measure (word scores) at +10 dB SNR and –15 dB SNR for some of the post hoc comparisons.

Although the study was designed to detect differences between three pens, one pen, and no pen, and statistically powered to detect differences of only 4 dB or greater, the seemingly poor performance of “1 pen” compared to “no pen” cannot be completely ignored. There was a small, but not statistically significant, improvement of 1.7 dB in the SRT, which would still represent a clinically significant improvement in speech perception. The “1 pen” condition was important to test because currently this configuration is the most likely way in which an adult would use the Roger system. However, it highlights that it is important to counsel listeners to have speakers hand around the Roger Pen rather than just placing it on the table. Microphone directionality is also a factor because the majority of subjects (9/12) were already using the T-mic when listening in the “no pen” condition. Because of the T-mic’s location at the entrance to the ear canal, a directional advantage already exists compared to an omnidirectional processor located on the top of the processor. Recall that the Roger transmitter in the “1 pen” condition functions in an omnidirectional mode at noise levels up to 65 dB but in a beam-forming mode at noise levels above 70 dB. Thus, at higher noise levels, the Roger Pen may have favored understanding the Talker in front of the listener, but the talkers more to the left and right may have already been to some extent outside of the beamformer.

Previous studies comparing Roger with conventional FM systems have shown that, at higher noise levels (70–80 dB A), the digital Roger is better than even other adaptive gain analog FM systems [11, 21]. This difference may be a consequence of the higher levels of maximum gain in the Roger (up to 30 dB), the greater bandwidth increasing the available speech cues, or a clearer overall signal because of less interference resulting from Roger’s frequency-hopping approach. In this study, the noise levels required to reach the SRT ranged from between 58 to 65 dB for one pen and from 71 to 78 dB for three pens. Thus, the noise levels were at the minimum level where a significant advantage for an adaptive digital system has been shown over a conventional fixed gain or analog dynamic gain system [9, 11, 21]. Nonetheless, all of these noise levels in this study were sufficient to reach the point where the +10 dB of gain starts to increase dynamically, which is important to consider particularly because a previous study with the Advanced Bionics Auria processor showed that a gain setting of greater than +10 produced the optimum results [22].

Note that the results in this study represent “good” performers (on average subjects performed at ceiling levels on sentences in quiet and had an average SRT with the processor alone of +5 dB). In addition, the Naida CI Q70 processor has a wide input dynamic range (80 dB), which can have an effect on the performance of sound processors with FM systems [22]. However, it is remarkable to notice that the use of adaptive digital technology can allow people with a CI to even surpass the performance of normally hearing individuals in noise [21].

From a use-case standpoint, the integrated Roger receiver 17 makes it easy for Naida CI Q70 users to access Roger technology and removes some of the complexity which may deter CI recipients from using an FM. Thus, more clinicians may be inclined to recommend and fit Roger to their patients using Advanced Bionics implants [8]. It would be interesting in future studies to compare adaptive digital transmission with other types of microphone directionality available on the latest sound processor models.

## Conclusions

The Roger Pen adaptive digital wireless transmission system and integrated Roger 17 receiver provided significant speech perception advantage in noise for users of a Naída CI Q70 sound processor. When used in a multi-talker network (MTN), there was a large statistically and clinically significant improvement, with an average gain in SRT of 14.8 dB over using an everyday microphone, and a 13.1 dB improvement over using a single Roger Pen. Clinicians should consider recommending a Roger system for CI recipients who need to communicate frequently in noisy environments.
